# Data generated from three quantitative mass spectral methods for the analysis of trivalent influenza vaccine antigens are compared

**DOI:** 10.1016/j.dib.2016.08.035

**Published:** 2016-08-25

**Authors:** Daryl G.S. Smith, Geneviève Gingras, Yves Aubin, Terry D. Cyr

**Affiliations:** Centre for Biologics Evaluation, Biologics and Genetic Therapies Directorate, Health Products and Food Branch, Health Canada, Ottawa, ON, Canada

**Keywords:** Protein quantitation, Influenza, Hemagglutinin, QConCAT, Hi3, Optimization

## Abstract

Herein we present the data necessary for generation of alternative means to produce equimolar mixtures of peptides (“Design and Expression of a QconCAT Protein to Validate Hi3 Protein Quantification of Influenza Vaccine Antigens” (D.G.S. Smith, G. Gingras, Y. Aubin, T.D. Cyr, 2016) [1]), such as QConCAT (“Trends in QconCATs for targeted proteomics” (J. Chen, I.V. Turko, 2014) [2] , “Natural flanking sequences for peptides included in a quantification concatamer internal standard” (C.S. Cheung, K.W. Anderson, M. Wang, I.V. Turko, 2015) [3]) and SpikeTides versus the label free Hi3 approach. The experimental design and the interpretation of results are discussed in the original article [1].

**Specifications Table**

**Table:** 

Subject area	*Chemistry*
More specific subject area	*Absolute quantitative proteome analysis*
Type of data	*Gene sequences, tables, graphs, links, fasta protein database*
How data was acquired	*Mass spectroscopy, QTOF (*Waters Synapt HDMS system (Milford, MA, USA)
Data format	*Analyzed*
Experimental factors	*The samples were digested with a higher than usual ratios of trypsin/substrate, source voltages optimized to reduce in-source fragmentation*
Experimental features	*The peptides were analyzed on a* using a reversed phase ( BEH130 C18) column with data independent MSMS then data analysis using PLGS 3.0 Waters Ltd.)
Data source location	*n/a*
Data accessibility	*Data is within this article*

**Value of the data**

• Excellent reproducibility of peptide concentration data produced by the analysis of tryptic digestions is the necessary for method comparisons. The costs associated with a particular approach in terms of reagents and time may be a pivotal factor.
• The Hi3 method is a very valuable approach in cases of relatively simple proteomes. The speed of method development for core laboratories can be critical. The Hi3 method is relatively straightforward, once reproducible results are obtained.
• The QConCAT design is very elegant but there are critical issues, such as the protein construct solubility and stability, which require careful attention to obtain optimal results. The database attached is necessary for analysis of the mass spectral results.

## 1 Data

The data are compiled in the bar graphs for the quantitative proteome analyses of trivalent influenza vaccines and standards using three methods: Hi3, QconCAT and synthetic peptides.

Databases contained either the customized QconCAT protein sequence or the full sequences of the proteins represented therein, along with trypsin, several human keratins (cRAP from http://www.thegpm.org/crap/) and, since the viruses are grown in chicken eggs, the entire chicken (*Gallus gallus*) proteome (ftp://ftp.ensembl.org/pub/current_fasta/gallus_gallus/pep/) as well as selected full length influenza proteins (mostly from GISAID http://platform.gisaid.org/epi3/frontend#4f5b25, and the World Health Organization http://www.who.int/influenza/vaccines/virus/en/.

## 2 Experimental design, materials and methods

Data was obtained by the comparison of mass spectral signal from the three most intense fully tryptic peptides identified from the samples versus the internal standard protein. Experiments were designed to compare the mass spectral signal strengths from equimolar tryptic peptides identified by the Hi3 method by five different QconCAT designs as well as from synthetic peptides. The samples analyzed were commercial trivalent influenza vaccines as well as monovalent influenza reference standards. The mass spectra were obtained by reversed phase separation using a C18 UPLC column, Waters nanoAcquity UPLC, directly coupled to a Waters Synapt HDMS mass spectrometer. The mass spectrometer was programmed to carry out data-independent MSMS and incorporated a lock spray of glu-fibrinopeptide. The data was processed using Protein Lynx Global Server 3.0 for the identification of the three most intense peptides from a given protein identified in a custom database which has been attached. The intensities from all charge states of a peptide are included in the software calculations; however, the intensities resulting from in-source fragmentation and modified peptides must be added manually.

### 2.1 Codon optimized gene sequences

#### 2.1.1 QconCAT 1

CATATGGATGACGATGATAAACTGGTGAACGAACTGACCGAATTCGCGAAACTGGGCGAATACGGCTTCCAGAATGCACTGATCGTTCGTCATCTGGTTGACGAACCACAAAACCTGATTAAAGACGCATTTCTGGGCTCCTTTCTGTACGAATACTCTCGCGTTGTTGGTCTGTCTACCCTGCCTGAGATTTACGAAAAACTGCCACTGGTCGGTGGTCATGAAGGCGCAGGCGTCGTCGTTGGTATGGGCGAAAACGTGAAATCCATCAGCATTGTAGGTTCCTACGTTGGCAACCGTGCCAATGAACTGCTGATCAACGTCAAATCCACCCAGAACGCGATCGACGAGATTACGAATAAAATGAACTACTACTGGACCCTGGTTGAGCCGGGCGATAAAGAACAGCTGTCCAGCGTGTCCTCTTTCGAACGTATGAACACGCAGTTTACCGCTGTAGGCAAAAGCACCCAGGCTGCCATCGACCAGATCAACGGTAAAATCGATCTGTGGTCCTATAATGCCGAGCTGCTGGTTGCCCTGGAAAATCAGCACACTATTGACCTGACTGATAGCGAAATGAACAAGGAGTTCTCTGAAGTGGAAGGTCGTTGGGACCTGTTCGTGGAACGTCTGTCTGGCGCGATGGACGAACTGCACAACGAAATCCTGGAACTGGATGAGAAACTGTCTACTCACAACGTTATCAACGCGGAAAACGCTCCGGGTGGCCCGTACAAAATTGTGGTGGACTACATGGTGCAGAAGAACCTGAACTCCCTGAGCGAGCTGGAAGTGAAAACTTTCTTCCTGACTCAGGGTGCCCTGCTGAACGACAAATACAACGGCATCATTACGGACACCATCAAATATGGTAACGGTGTTTGGATCGGTCGCGGTGACGTGTTCGTTATCCGCACGCTGCTGATGAACGAGCTGGGTGTTCCGTTCCACCTGGGCACCAAGCTGGTGGATTCTGTAGTCTCTTGGAGCAAAGTAATCGAGGGCTGGAGCAACCCGAAGATTCTGTTTATTGAAGAAGGCAAAGGTGTAACCCTGCTGCTGCCGGAACCGGAATGGACCTACCCGCGCCTGAACGTTGAAACTGATACTGCGGAAATTCGTTATGGTGAAGCGTATACCGACACCTATCACAGCTACGCTAACAAAGAGTGGACCTATATCGGTGTTGATGGTCCGGATAACAACGCTCTGCTGAAAGGCGGTCTGGAACCTATCAACTTCCAAACCGCAGCGGACCAGGCGCGTATCTCTCAAGCAGTACACGCTGCGCACGCAGAAATCAACGAAGCTGGCCGTCTGACCGAGTGGACTTCTTCCAATGTTATGGAAGAACGTAACGTTCTGCAGCCGTCTTCCGTAGATTCTCAGACCGCAATGGTTCTGGTAAACGCTATCGTTTTCAAGTAAGGATCC.

#### 2.1.2 QconCAT 2

CATATGGACGATGACGACAAGCATGTCAAACTGGTGAACGAACTGACTGAATTCGCAAAAACTTGCGTGCGTTTCGAAAAACTGGGCGAATACGGTTTCCAGAACGCACTGATCGTGCGTTACACCCGTAAACTGAAACACCTGGTGGATGAACCTCAAAACCTGATCAAACAGAACTGCCGTCGTCCAATCAAAGTAGTGGGTCTGTCTACCCTGCCGGAGATTTACGAAAAAATGGAAAAGCGTCCGGTGAAGCTGCCTCTGGTCGGCGGTCACGAAGGCGCTGGTGTTGTTGTTGGTATGGGCGAGAACGTGAAAGGTTGGAAGGTTGTTAAATCTATTAGCATCGTTGGCTCTTACGTAGGTAACCGCGCCGACACTCGTGATCTGAAGAGCACCCAGAATGCCATCGATGAAATCACCAACAAAGTCAACTCTCGCGAAGGTCGTATGAACTACTATTGGACCCTGGTTGAACCGGGCGACAAGATTACCTTTCGTGAACTGCGCGAACAGCTGTCTTCTGTGTCCAGCTTCGAACGTTTTGAAATCCGTGAATGCCGCACGTTCTTTCTGACGCAGGGTGCACTGCTGAACGACAAACATTCCAACCGCGTACTGAAATACAACGGTATCATCACTGATACCATTAAATCCTGGCGCAGCTTTAAATACGGTAACGGCGTCTGGATCGGTCGTACGAAATCTGACCTGAAATCCACCCAGGCGGCCATTGATCAGATCAACGGCAAACTGAACCGTGATACCAAAATCGATCTGTGGTCCTACAACGCGGAACTGCTGGTGGCCCTGGAAAACCAACACACCATCGACCTGACCGACTCTGAAATGAACAAACTGTTCGAGCGTATTGAAAAAGAATTTTCCGAGGTGGAGGGTCGCATCCAAGATCGTCGCCCGTACCGCACTCTGCTGATGAATGAACTGGGTGTTCCATTCCACCTGGGTACCAAACAGGTTTGCCGCAACGGCCGTCTGGTTGACAGCGTAGTAAGCTGGTCTAAAGAAATCCTGCGTACCTTCAAAGTTATCGAAGGTTGGTCTAACCCGAAATCTAAACTGCTGCAGCGCCTGTCTGGTGCGATGGACGAACTGCACAACGAGATCCTGGAACTGGATGAGAAAGTTGACGACCGTCACATCCGCCTGTCCACCCACAATGTAATTAACGCAGAAAACGCTCCGGGCGGTCCGTACAAAATTGGTACTCGCTCCGGTCGTATTGTGGTTGACTATATGGTACAGAAGTCTGGCAAAGCAACTAAAGGCGTTACGCTGCTGCTGCCGGAACCGGAATGGACTTATCCGCGTCTGTCCTGTCGTTTCGTCAAGCTGAACGTTGAAACCGACACGGCGGAAATTCGTCTGATGTGTAAAGTGAAATATGGCGAAGCGTATACCGATACTTACCATTCTTACGCTAACAAGATCCTGCGCCTGTATCGTGGCGGCCTGGAACCGATTAATTTCCAGACTGCGGCGGATCAGGCTCGTGAGCTGATCCGTAGCCTGAAAATCTCCCAGGCAGTACACGCTGCTCACGCTGAAATCAACGAAGCGGGCCGTGAGGTAGTTCGTTTCGAGAAACTGACCGAATGGACCAGCAGCAATGTTATGGAGGAGCGTAAAATCAAATAAGGATCC.

#### 2.1.3 QconCAT 3 and 4

CATATGGATGACGACGACAAAGCAAGCGGTAAACTGGTGAACGAGCTGACTGAGTTCGCTAAAGCGAGCGGTAAGCTGGGTGAATACGGTTTCCAGAACGCACTGATCGTACGTGCTTCTGGTAAACACCTGGTCGACGAGCCTCAAAACCTGATTAAAGCTAGCGGCAAAGACGCCTTCCTGGGTAGCTTCCTGTACGAGTACAGCCGTGCTTCTGGCAAAGTAGTAGGTCTGAGCACTCTGCCAGAAATCTACGAGAAAGCGTCTGGTAAGCTGCCTCTGGTCGGTGGTCATGAAGGTGCTGGTGTAGTAGTAGGTATGGGTGAGAACGTGAAAGCATCCGGTAAAAGCATCAGCATCGTCGGTTCCTACGTCGGTAACCGTGCAAGCGGTAAAGCTAACGAGCTGCTGATCAACGTCAAGGCAAGCGGCAAATCTACCCAGAACGCAATCGACGAGATCACTAACAAAGCATCTGGTAAAATGAACTACTACTGGACCCTGGTCGAGCCGGGCGACAAAGCCAGCGGTAAGGAGCAGCTGTCTTCTGTGTCTTCCTTCGAGCGTGCATCTGGTAAGATGAACACCCAGTTCACGGCAGTGGGTAAAGCATCCGGCAAGACTTTCTTCCTGACTCAGGGTGCACTGCTGAACGATAAAGCTTCCGGTAAATACAACGGCATCATCACGGACACCATCAAAGCGTCTGGCAAGTACGGCAACGGTGTGTGGATCGGTCGTGCTTCCGGTAAGGGTGATGTTTTCGTGATCCGTGCATCTGGCAAATCCACTCAGGCAGCAATCGACCAAATCAACGGTAAGGCTTCCGGCAAGATCGACCTGTGGTCTTACAACGCTGAACTGCTGGTTGCTCTGGAAAACCAGCATACTATCGACCTGACCGACTCTGAAATGAACAAAGCCTCCGGTAAAGAATTCTCCGAAGTGGAAGGCCGCGCGTCTGGTAAATGGGACCTGTTTGTGGAACGCGCCTCTGGTAAGACTCTGCTGATGAACGAACTGGGTGTGCCATTTCACCTGGGTACGAAAGCATCTGGCAAGCTGGTGGATTCTGTGGTATCCTGGTCTAAAGCCTCTGGTAAAGTTATCGAAGGCTGGTCCAACCCGAAAGCCAGCGGCAAGATCCTGTTTATTGAAGAAGGTAAAGCGTCCGGTAAACTGTCCGGCGCGATGGACGAACTGCACAACGAAATTCTGGAACTGGATGAAAAAGCTTCTGGCAAACTGTCCACCCACAACGTTATTAACGCGGAAAACGCCCCGGGCGGCCCGTACAAAGCTAGCGGTAAAATCGTTGTTGATTATATGGTTCAGAAAGCTTCCGGCAAAAACCTGAACAGCCTGTCCGAACTGGAAGTTAAAGCCTCCGGCAAAGGCGTTACCCTGCTGCTGCCGGAACCGGAATGGACCTACCCGCGTGCCAGCGGCAAACTGAATGTTGAAACCGATACCGCTGAAATTCGCGCCAGCGGTAAATATGGCGAAGCTTATACCGATACCTATCACTCCTATGCGAACAAAGCGAGCGGCAAAGAATGGACCTATATTGGCGTAGATGGCCCGGATAACAACGCGCTGCTGAAAGCCTCTGGCAAAGGCGGCCTGGAACCGATTAATTTTCAGACCGCGGCTGATCAGGCTCGTGCGAGCGGTAAAATTTCTCAGGCGGTTCATGCGGCGCACGCGGAAATTAATGAAGCGGGCCGTGCGTCTGGCAAACTGACCGAATGGACGTCTTCTAATGTTATGGAAGAACGCGCATCCGGCAAAAATGTTCTGCAACCGTCTAGCGTTGATTCCCAGACCGCGATGGTTCTGGTTAATGCGATTGTTTTCAAAGCGTCCGGCAAATAAGGATCC.

#### 2.1.4 QconCAT 5

CATATGGCTGGTCGTGCGTCTGGTAAACTGGGTGAGTACGGTTTTCAGAACGCGCTGATCGTACGTGCGTCTGGTAAAGTAGTCGGTCTGTCTACCCTCCCGGAGATCTACGAAAAAGCGTCCGGTAAAGAGGTCCTGGTTCTGTGGGGTATTCACCACCCGTCTACTTCTGCAGATCAGCAGTCTCTGTACCAGAACGCAGACGCTTACGTATTCGTTGGCTCTTCTCGTGCGTCTGGCAAAATCGACCTGTGGTCTTACAACGCCGAACTGCTGGTTGCCCTGGAAAACCAGCACACTATCGACCTGACCGACTCCGAAATGAACAAAGCGTCCGGTAAACTGTCTGGTGCGATGGACGAACTGCACAACGAAATCCTGGAACTGGACGAGAAAGCCAGCGGTAAAACCTTCTTCCTGACTCAGGGTGCGCTGCTGAACGACAAAGCTTCTGGCAAAACCCTGCTGATGAACGAACTGGGTGTTCCGTTTCACCTCGGTACCAAAGCGTCTGGTAAAGGTGTTACCCTGCTGCTGCCGGAACCGGAATGGACTTATCCACGTGCCTCTGGTAAAGGTGGTCTGGAACCGATCAACTTTCAGACGGCCGCAGATCAGGCACGTGCTTCTGGTAAAATCTCTCAGGCTGTTCACGCCGCGCACGCAGAAATCAACGAAGCAGGTCGTGCTTCTGGCAAACTGAACGTTGAAACCGACACCGCGGAAATCCGTGCCTCTGGTAAACTGGTTGACAGCGTTGTTTCTTGGTCCAAAGCGTCCGGTAAATACAACGGTATCATCACCGACACCATCAAAGCCTCTGGTAAATTCACCTCCTCTGCCAACGGTGTTACGACCCACTACGTATCTCAGATCGGTGGTTTCCCGGATCAGACCGAAGACGGTGGTCTGCCGCAGTCTGGTCGTGCTTCTGGTAAATCTACCCAGGCGGCGATTGACCAGATCAACGGTAAAGCGTCCGGCAAATCTACGCAGAACGCGATCGACGAGATCACCAACAAAGCCTCTGGTAAACTGCCGCTGGTAGGTGGTCACGAAGGTGCAGGTGTTGTAGTGGGTATGGGTGAGAACGTGAAAGCGAGCGGTAAACTGGTTAACGAACTGACGGAGTTCGCCAAAGCCTCTGGTAAAAACCTGAACAGCCTCAGCGAACTGGAAGTGAAAGCCTCTGGCAAATACGGTAACGGTGTTTGGATCGGTCGTGCTAGCGGTAAATCTGGTTACAGCGGTATCTTCTCTGTTGAGGGTAAAGCCTCCGGTAAATACGGTGAAGCCTACACGGATACCTACCACTCTTACGCCAAAGCGAGCGGTAAACTGACCGAATGGACGAGCTCTAACGTTATGGAGGAACGTGCCTCTGGTAAAGACGCTTTCCTGGGCTCCTTCCTGTACGAATACTCTCGTGCGTCTGGTAAATCTATCTCTATCGTCGGCTCCTACGTAGGTAACCGTGCGTCTGGTAAAATGAACTACTACTGGACCCTGGTTGAACCGGGTGACAAAGCTTCCGGTAAATCTCAGCAGGCGGTTATCCCGAACATCGGTTTTCGTCCACGTGCTTCTGGCAAACTGAACTGGCTGACCCACCTGAACTTCAAAGCCTCCGGTAAAATGAACACCCAGTTCACGGCGGTTGGTAAAGCGTCTGGTAAAGCCAACGAACTGCTGATCAACGTGAAAGCCTCCGGTAAACACCTCGTAGACGAACCGCAGAACCTGATCAAAGCGTCTGGCAAAAACGTACTCCAGCCGTCTTCTGTTGACTCTCAGACCGCTATGGTTCTGGTCAACGCGATCGTATTCAAAGCCAGCGGTAAAGGTAACTCTGCCCCGCTGATCATTCGTGCCTCTGGTAAAGGTTGGGCTTTTGACGACGGTAACGACGTTTGGATGGGTCGTGCGTCTGGTAAAGGCGACGTATTCGTTATCCGTGCGTCTGGTAAAGCGGACACCATCTCTTCTCAGATCGAACTGGCCGTTCTGCTGTCTAACGAGGGTATCATCAACTCCGAAGACGAGCACCTGCTGGCTCTGGAACGTGCCTCTGGTAAACTGGCAGCGGCCCTGGAACACCACCACCACCACCACTAATAGGGATCC.

### 2.2 Peptide Sequences

#### 2.2.1 QconCAT 1-4

**Table:** 

**Protein**	Peptides (*sequence omitted in QconCAT 2)
BSA – Bovine Serum Albumin	LVNELTEFAK
	LGEYGFQNALIVR
	HLVDEPQNLIK
	DAFLGSFLYEYSR*
ADH – Alcohol Dehydrogenase (*Saccharomyces cerevisiae*)	VVGLSTLPEIYEK
	LPLVGGHEGAGVVVGMGENVK
	SISIVGSYVGNR
	ANELLINVK*
H1 – Hemagglutinin (H1N1)	STQNAIDEITNK
	MNYYWTLVEPGDK
	EQLSSVSSFER
	MNTQFTAVGK*
N1 – Neuraminidase A/California (H1N1)	TFFLTQGALLNDK
	YNGIITDTIK
	YGNGVWIGR
	GDVFVIR*
H3 – Hemagglutinin A/Victoria (H3N2)	STQAAIDQINGK
	IDLWSYNAELLVALENQHTIDLTDSEMNK
	EFSEVEGR
	WDLFVER*
N2 – Neuraminidase A/Victoria (H3N2)	TLLMNELGVPFHLGTK
	LVDSVVSWSK
	VIEGWSNPK
	ILFIEEGK*
HB – Hemagglutinin B/Brisbane	LSGAMDELHNEILELDEK
	LSTHNVINAENAPGGPYK
	IVVDYMVQK
	NLNSLSELEVK*
NB – Neuraminidase B/Brisbane	GVTLLLPEPEWTYPR
	LNVETDTAEIR
	YGEAYTDTYHSYANK
	EWTYIGVDGPDNNALLK*
Oval – Ovalbumin (*Gallus gallus*)	GGLEPINFQTAADQAR
	ISQAVHAAHAEINEAGR
	LTEWTSSNVMEER
	NVLQPSSVDSQTAMVLVNAIVFK*

#### 2.2.2 QconCAT 5 and SpikeTides

**Figure:**
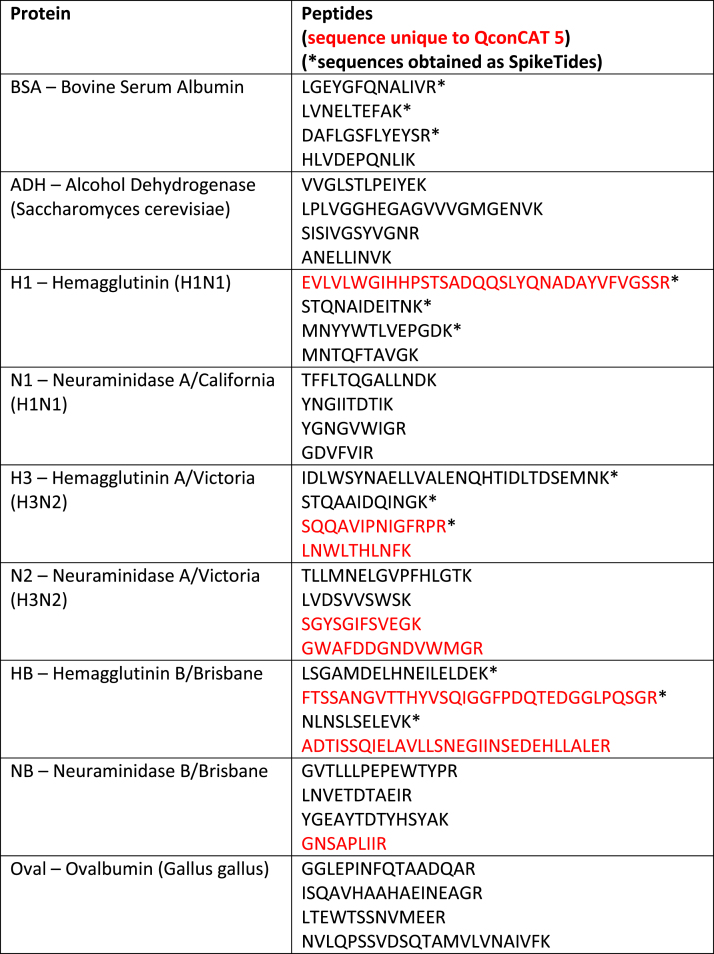

**Figure:**
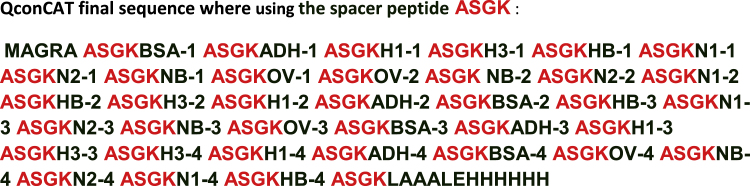

.

**Figure:**
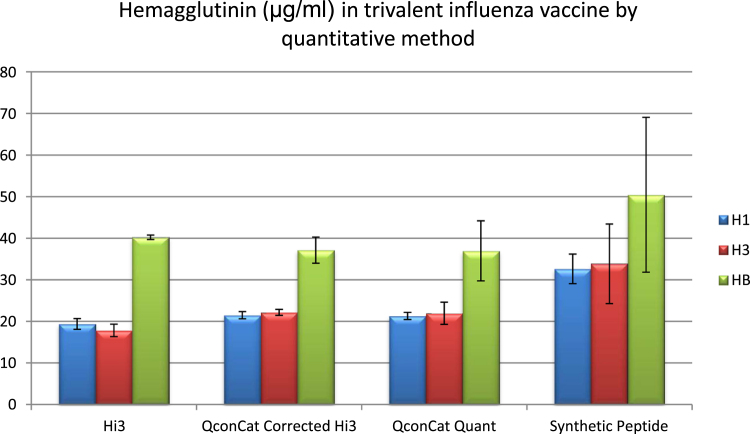

.

**Figure:**
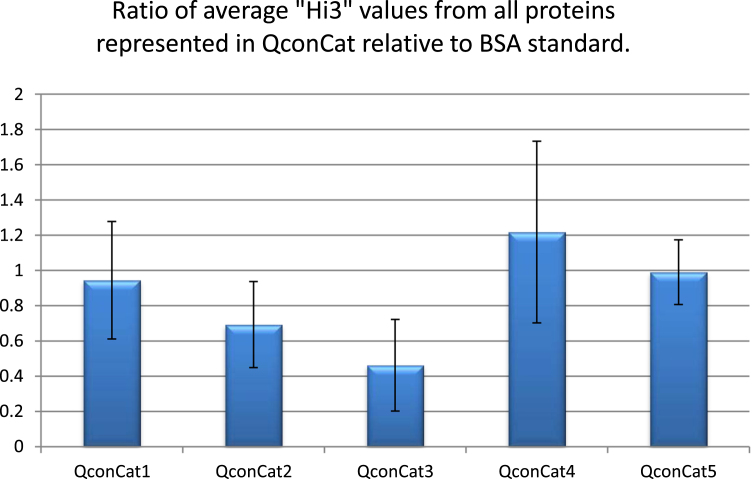

**FASTA Protein Database used: 2015_2016_FluQuant.fasta**
